# Organ donation after euthanasia starting at home: a mixed-methods study of preliminary nationwide Dutch experiences

**DOI:** 10.3389/ti.2026.16452

**Published:** 2026-09-14

**Authors:** Isabelle Manders, Nathalie van Dijk, Johan Sonneveld, Wim de Jongh, Jan Bollen, Nichon E. Jansen, Walther van Mook

**Affiliations:** 1 Faculty of Health, Medicine & Life Sciences, Maastricht University, Maastricht, Netherlands; 2 Department of Intensive Care Medicine, Maastricht University Medical Center+, Maastricht, Netherlands; 3 Department of Anaesthesiology and Intensive Care Medicine, Isala Hospital, Zwolle, Netherlands; 4 Department of Surgery, Transplantation Coordination Services, Maastricht University Medical Center+, Maastricht, Netherlands; 5 Department of Anaesthesiology, Pain and Palliative Medicine, Radboud University Medical Center, Nijmegen, Netherlands; 6 Department of Policy and Research, Dutch Transplant Foundation, Leiden, Netherlands; 7 Academy for Postgraduate Training, Maastricht University Medical Center+, Maastricht, Netherlands; 8 School of Health Professions Education, Maastricht University, Maastricht, Netherlands

**Keywords:** case series, medical aid in dying, national survey, ODEH, Organ Donation after Euthanasia starting at Home

## Abstract

Organ Donation after Euthanasia starting at Home (ODEH) initiates the euthanasia trajectory at home with deep sedation, followed by transfer to the hospital for completion of euthanasia and organ procurement. We evaluated all eight Dutch ODEH procedures up to February 2024 using a mixed-methods design: descriptive analysis of anonymized case data (Dutch Transplant Foundation and hospital records) and semi-structured interviews (November 2023–February 2024) with involved professionals. All procedures were performed in three hospitals. Patients had neurodegenerative disease (n = 6) or psychiatric disorders (n = 2) and were aged from the mid-30s to the early-70s (mean 50.4 years). Professionals highlighted strict separation of euthanasia and donation decisions, time-critical coordination between home and hospital, variation in sedation/anesthesia and transport modality (regular ambulance vs. mobile ICU), regional constraints in staffing and infrastructure, emotional impact on professionals, and registry/coding considerations (including HSMR). ODEH is feasible within the existing Dutch legal framework and is valued for enabling a home farewell without compromising donation. A flexible practice manual defining core safeguards and decision points may support consistent implementation.

## Graphical Abstract

**Figure:**
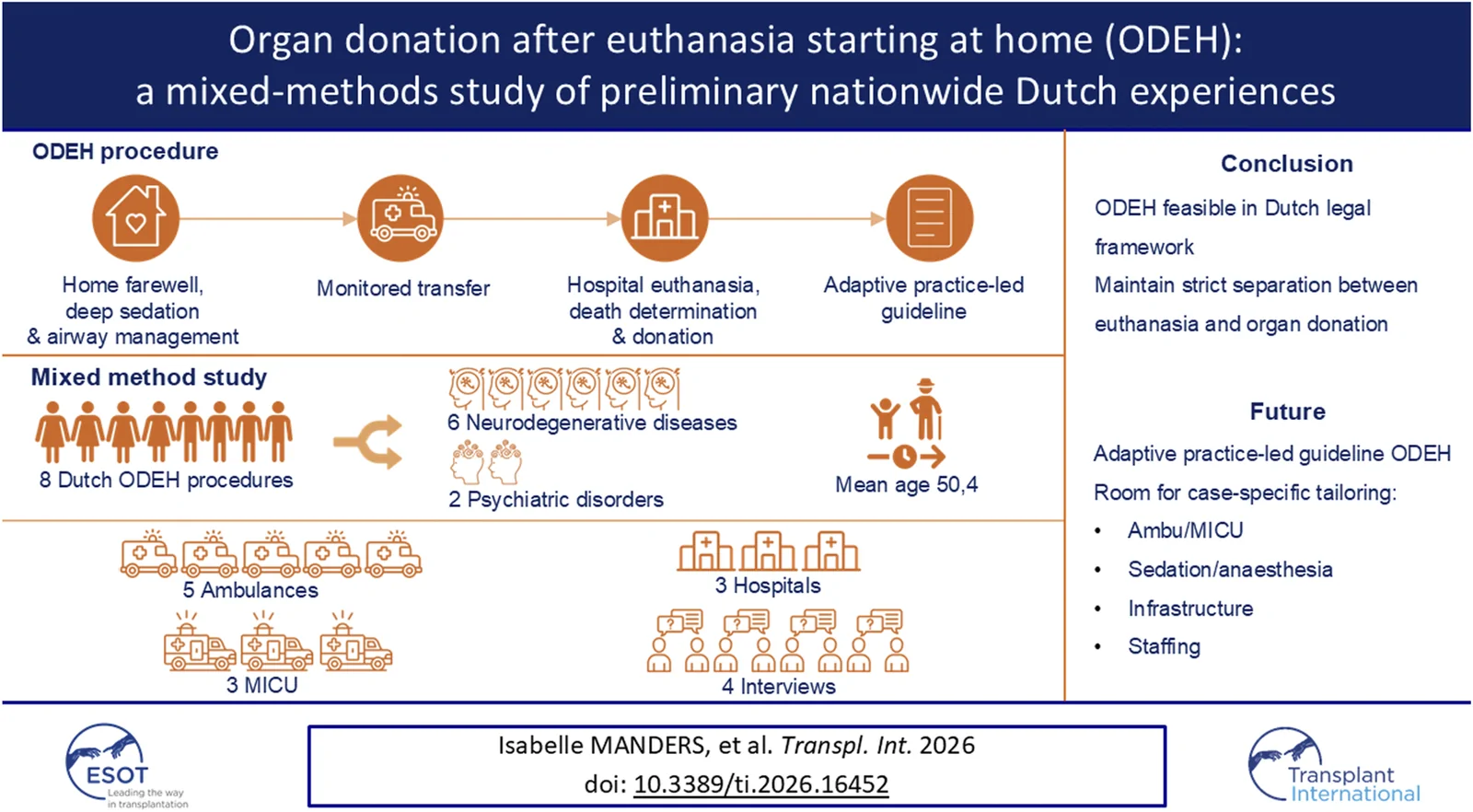

## Introduction

Organ Donation after Euthanasia (ODE) and Organ Donation after Euthanasia starting at Home (ODEH) combine two sensitive medical practices: euthanasia and organ donation. Since 2012, Dutch ODE initially involved patients with neurodegenerative disease [1] and later expanded to patients suffering unbearably from psychiatric disorders and (early stages of) dementia [2–4]. Currently, more than 180 patients in the Netherlands have chosen ODE. In conventional ODE, euthanasia, death determination, and subsequent organ procurement are performed in a controlled hospital setting.

Since 2017, up to October 2025, thirteen ODEH procedures have been performed, a personalized pathway in which the euthanasia trajectory is initiated at home, typically requested by patients who do not wish to undergo a hospital-based ODE procedure, but instead prefer to say farewell in their home environment. In this process, the patient is deeply sedated at home, the airway is secured, and mechanical ventilation is started, followed by transporting the patient to the hospital for the final administration of euthanasia medication and organ donation [5, 6]. ODEH allows patients to say goodbye in a familiar environment and to avoid the conscious experience of hospital admission, which supports the patient’s autonomy and dignity without necessarily compromising organ donation potential. At the same time, ODEH introduces additional practical, ethical, and legal challenges, including time-critical coordination between home and hospital teams and questions about out-of-hospital airway management and transport [7, 8].

ODEH is currently only generally addressed in the national Dutch Transplant Foundation (NTS) guideline on ODE [9, 10]. Although several publications have discussed ODEH, a systematic analysis of Dutch cases is lacking, and a focused, practice-oriented manual as an addendum to the national guideline is still absent [2, 11].

We therefore examined Dutch ODEH procedures to identify shared elements and variation, and to describe practical, ethical, and legal challenges. These findings may inform an ODEH manual with recommendations that support flexible, patient centered care while enabling consistent, legally robust implementation across hospitals in the Netherlands and other countries where ODEH is or may become legally permissible.

## Materials and methods

A mixed-methods design was applied to explore practical, ethical and legal aspects of ODEH.

### Quantitative data: case data

We included all ODEH procedures performed in the Netherlands up to February 2024. Data from the Dutch Transplant Foundation (NTS) and hospital records were anonymized and included demographics (age, sex), relevant clinical characteristics (diagnosis, comorbidities) and logistical/procedural details (treating hospital, mode of transport and pathway characteristics). These data were used to explore similarities and differences in practice between hospitals and regions.

### Qualitative data: individual semi-structured interviews

Individual semi-structured interviews were conducted between November 2023 and February 2024 with healthcare professionals who had participated in at least one ODEH procedure. Purposeful sampling ensured direct procedural experience. Interviews (≈90 min) followed a semi-structured guide developed with experts in ODE(H) practice (Addendum 1) and covered the full pathway: from the initial request and approvals to home sedation, airway management, transfer logistics (regular ambulance/MICU), in hospital completion of euthanasia, organ procurement and post-procedure evaluation. Practical, ethical and legal considerations were explicitly explored.

### Ethical approval and consent

The study was conducted in accordance with relevant ethical standards [12, 13]. Quantitative analysis was approved by the Medical Ethics Review Committee (METC 2025-0477). For the qualitative component, verbal informed consent was obtained before each interview. Participants were informed about the study purpose, voluntary participation, data use and their right to withdraw at any time, without obligation to provide a reason.

### Analysis

#### Quantitative analysis

Data were analyzed descriptively in SPSS (version 28).

#### Qualitative analysis

Interviews were transcribed (edited in Microsoft Word) and analyzed using thematic analysis. We followed AMEE guidance for thematic analysis and reported according to COREQ criteria [14, 15]. The primary researcher performed initial coding; a second researcher reviewed and refined codes and themes. Member checking was performed by sharing interview summaries with participants. Formal data saturation could not be established because only eight ODEH procedures had occurred by the end of the study period, limiting the pool of eligible healthcare professionals.

## Results

We included eight ODEH procedures performed in the Netherlands up to February 2024. Procedures were conducted in three hospitals: two University Medical Centers (UMCs) and one large teaching hospital (STZ), all located in the eastern regions of the Netherlands.

### Quantitative data

Patient characteristics are summarized in Table 1.

**TABLE 1:** Overview of the ODEH cases’ demographic and clinical data.

Patient	Hospital	Geographic region of Netherlands	Gender	Age category	Diagnosis	Mode of transport	Euthanasia providing physician
A	Hospital C: Large Teaching hospital	North-East	Male	40–50	Amyotrophic lateral sclerosis (ALS)	MICU	General practitioner
B	Hospital A: University medical Center A	East	Female	50–60	Amyotrophic lateral sclerosis (ALS)	MICU	Hospital specialist (neurologist)
C	Hospital B: University medical Center B	South	Male	N/A	Multi system atrophy (MSA)	MICU	General practitioner
D	Hospital C: Large Teaching hospital	North-East	Male	70–80	Early-stage dementia, pain syndrome, accumulation of health problems related to age	Regular ambulance	General practitioner
E	Hospital C: Large Teaching hospital	North-East	Female	50–60	Huntington’s disease	Regular ambulance	Elderly care physician
F	Hospital C: Large Teaching hospital	North-East	Male	50–60	Amyotrophic lateral sclerosis (ALS)	Regular ambulance	Elderly care physician
G	Hospital C: Large Teaching Hospital	North-East	Female	30–40	Psychiatric disorder	Regular ambulance	Psychiatrist
H	Hospital C: Large Teaching hospital	North-East	Female	30–40	Psychiatric disorder	Regular ambulance	Psychiatrist

Six patients suffered from neurodegenerative disease, and two from psychiatric disorders. Ages ranged from the mid-30s to the early 70s (mean 50.4 years; range ±13.4), with a balanced gender distribution.

### Qualitative data

Four semi-structured interviews were conducted with professionals directly involved in ODEH, including organ donation coordinators, an anesthesiologist and intensive care specialists, each from a performing centre and closely involved in the ODEH procedures. Analysis identified recurring themes: safeguarding patient autonomy and role separation between euthanasia and donation; logistical coordination between home and hospital settings; multi- and interdisciplinary collaboration; transport modalities; regional differences in resource availability; sedation/anesthesia practice; emotional impact on staff; and institutional reporting (including HSMR considerations).

### The ODEH process in general

ODEH typically started when patients explicitly expressed a wish for organ donation during advance care planning, months (case F) or even years (cases A, C, D and E) before euthanasia. After the euthanasia request was assessed by an independent SCEN physician (Support and Consultation on Euthanasia in the Netherlands), the patient’s donation wish was revisited. Separate teams remained responsible for euthanasia and organ donation to prevent potential conflicts of interest and undue influence on the patient’s decision.

After approval, ODEH preparations focused on aligning timing, transport, staff and equipment across care settings. On the day of the procedure, the euthanasia-providing physician reconfirmed the patient’s decision, allowed time for farewell, and initiated euthanasia-related sedation at home. Administration of the initial sedative (oral or intravenous) marked a procedural point of no return within the euthanasia process. Following the establishment of adequate sedation (typically after loss of consciousness), the anesthesiologist, whether already present or entering upon request, initiated general anesthesia to preserve procedural and role separation. If the initial sedative effect was insufficient (e.g., suspected benzodiazepine resistance), the anesthesiologist could advise on additional agents, while administration remained the responsibility of the euthanasia-providing physician. After induction of general anesthesia, the airway was secured, and mechanical ventilation was initiated while monitoring vital functions. The patient was transported to the hospital, where the euthanasia medication was administered on the ICU and ventilation was withdrawn. Death was determined after circulatory arrest and respecting the legally obligatory five-minute no-touch period by the euthanasia-providing physician, with support from the intensivist for monitoring and interpretation when intra-arterial monitoring was used. The forensic physician/municipal coroner and public prosecutor were likewise consulted according to Dutch legally mandated procedures for euthanasia and donation after non-natural death; organ recovery commenced only after the required permission regarding formal post-mortem release of the body had been obtained. In line with euthanasia practice, the euthanasia-providing physician did not leave the patient after initiation of sedation and therefore accompanied the patient during transport, ensuring continuity of responsibility throughout the home-to-hospital phase.

### ODEH-specific challenges

#### Ethical challenges regarding patient autonomy

Professionals consistently emphasized that, as in conventional ODE, decisions about euthanasia and organ donation must remain clearly separated to safeguard voluntariness. Home-based sedation was administered only after reconfirmation of the wish to proceed and after the patient had said farewell. This step was considered central to dignity in ODEH because it ensured that all awareness was lost before any hospital-based procedures.

Participants described actively guarding against any perception that organ donation could influence the euthanasia decision. This was achieved through strict role delineation between professionals involved in euthanasia and those responsible for organ donation, repeated reconfirmation of the patient’s autonomous wish, and transparent communication with patients and relatives about the voluntary nature of both decisions. These safeguards were considered essential to prevent both actual and perceived pressure. Although some patients had previously expressed a desire to donate organs months or even years before the final procedure, the key safeguard was that the assessment of euthanasia eligibility and the decision to proceed were made independently of the donation request, ensuring that organ donation could not serve as a driver, incentive, or influencing factor in the euthanasia decision.

#### Logistical coordination between home and hospital activities

Two organizational models were used. Earlier cases were typically coordinated within local hospital structures (local coordination, staffing and transport). Later cases could be facilitated by applying a nationally coordinated external anesthesia and transport support model, enabling centers with limited ODEH experience to use the latter pathway while maintaining the same core safeguards. Participants regarded this as a difference in logistical feasibility rather than in ethical or legal principles.

Patient G illustrated how logistics can be challenging in non-urban settings. Because of distance-dependent constraints, staffing, monitoring/transport capacity, and hospital readiness (including ICU and operating room availability) had to be aligned early and flexibly. This case suggested that ODEH can remain feasible in non-urban settings when teams plan pragmatically around what is necessary for safe transfer and for an individual patient, rather than around a single fixed institutional model.

#### Multi-and interdisciplinary collaboration

Professionals repeatedly highlighted the need for close collaboration between home care providers, hospital teams and organ donation services. Longer travel distances, limited MICU availability (cases B and C) and limited on-site resources in some hospitals (e.g., on-call anesthesiology, donation-coordinator coverage, ICU/OR capacity) could prolong planning.

Patient E illustrated how planning was tailored to preserve privacy while enabling donation. She wished to avoid being conscious during hospital diagnostics and to minimize exposure to hospital staff in her final weeks, while remaining strongly motivated to donate. A bespoke pathway was arranged: early morning admission with her parents to a private, non-active ICU unit, where the elderly-care physician (also the euthanasia-providing physician) administered 10 mg intravenous midazolam, after which she lost consciousness. The anesthesiologist then deepened sedation to general anesthesia, secured the airway and initiated mechanical ventilation. All pre-operative investigations were completed under continuous anesthesia. Pending organ allocation, she remained anaesthetized in the ICU under the attendance of her euthanasia-providing physician, and euthanasia was eventually performed mid-evening. Because sedation/anesthesia was initiated in a private, controlled hospital setting rather than at home, this case should not be classified as true ODEH. This variant can be interpreted as an intermediate pathway between conventional ODE and ODEH; Organ Donation after Euthanasia with Anesthesia in a Hospital Setting (ODE-AHS). This approach may offer an alternative when home-based sedation/anesthesia is not feasible, while still avoiding conscious exposure to the usual hospital-based donation pathway.

#### Mode of transport: MICUs versus regular ambulances

Transport modality was chosen pragmatically based on availability, local infrastructure and clinical risk assessment (e.g., anticipated airway/ventilation difficulty and hemodynamic stability). Because the airway was secured before transfer, the key transport risks were tube displacement/ventilation failure and maintaining physiological stability. Teams therefore used a “right-sizing” approach, matching monitoring and staffing to the expected risk profile and transit characteristics rather than defaulting to maximal resources; interviewees stressed that ODEH patients are not ICU patients in the conventional sense.

Both regular ambulances and mobile intensive care transport services were used. Regular ambulances were considered appropriate when cardio-respiratory risk was assessed as low, the airway was secured, and adequate monitoring and ventilation support were available. Mobile intensive care transport was used when additional staffing, monitoring capacity, or organizational feasibility were required; MICU configurations varied regionally and could sometimes be selected for practical reasons (e.g., seating configurations that enabled accompaniment). Participants reported regional differences in MICU coverage, on-call anesthesia availability and coordination capacity. UMCs generally had large, pre-existing networks and dedicated donation teams, which initially impacted the relative planning time. By contrast, teaching hospitals showed high flexibility in ODEH performance, drawing on experience in acute (transport) services after the first ODEH, subsequently enabling ODEH within 1 week.

#### Nature and performance of sedation

A defining feature of ODEH is deep sedation at home prior to transfer, in contrast to conventional ODE, in which the euthanasia medication, including the coma-inducing agent, is administered in the hospital. Across cases, home sedation was consistently applied, but centers differed in the practical support they provided and in how they organized responsibilities and risk assessment. Administration of the initial euthanasia-related sedation remained the responsibility of the euthanasia-providing physician. Centre-level variation mainly concerned route (oral and/or intravenous), regimen (e.g., oral premedication and/or intravenous midazolam with optional titration) and contingency planning. When reduced responsiveness to benzodiazepines was anticipated (for example, due to chronic benzodiazepine use or psychopharmacological medication), consultation took place between the euthanasia providing physician and the anesthesia team regarding alternative or additional agents, while responsibility remained with the euthanasia providing physician. Interviewees also noted that similar clinical conditions could be labelled “high” or “low” risk across centers, reflecting differences in experience and risk perception.

#### Emotional and psychological support for healthcare providers

Participants described ODEH as emotionally and organizationally demanding. Stressors included time-critical planning, performing anesthesia and airway management outside the hospital, the transition from end-of-life care to donation, and the long duration of the ODEH trajectories, which could lead to strong bonds with patients and families. This required medical expertise, coordination skills and emotional resilience. Advance planning therefore also included identifying professionals willing to participate in the ODEH pathway. In line with Dutch professional guidance, staff with conscientious objections to euthanasia or to involvement in organ donation after euthanasia could refrain from participation; staffing for transport, organ recovery and transplantation was arranged accordingly [9].

#### Institutional reporting and HSMR statistics

Professionals raised concerns that ODEH (as well as conventional ODE) could affect hospital standardized mortality ratio (HSMR) metrics because these patients are registered as hospital deaths. Interviewees considered this primarily a registration issue. In the National ICU Evaluation (NICE) registry, admissions related to post-mortem donation pathways can be coded as “admission solely for organ donation” (i.e., deceased during admission; admission for donation only). Participants reported applying similar coding for ODE and ODEH, thereby limiting the potential impact on ICU mortality statistics.

### ODEH commonalities and differences

Across cases, professionals described a consistent focus on patient-centered care, but pathways differed in governance structures, approval sequencing, and how patient/family needs were addressed. Ethics committee involvement was not described as mandatory in all cases, but was reported particularly during initial implementation of ODEH within a hospital or in novel cases. They also described greater flexibility in managing logistical and financial constraints, for example by combining pre-donation work-up with planned ICU/OR capacity, clustering or minimizing preparatory tests where possible, tailoring investigations to the patient’s burden and preferences, and using regular ambulances when clinically appropriate.

When relatives’ perspectives were reported by professionals, ODEH was described as positively impacting the patient and family experience by preserving a sense of control over timing and environment. One professional characterized ODEH as “a unique way to say goodbye, with added meaning through organ donation.” In several cases, professionals indicated that patients would likely not have opted for a conventional, hospital-based ODE procedure if the ODEH pathway had not been available.

Importantly, participants attributed identified differences primarily to local governance routes and sequencing of approvals, rather than to differences in overall diligence.

Some hospitals used a more formal organizational model, with detailed cross-departmental planning and early involvement of an organ donation coordinator and a donation intensivist. These roles facilitated planning (e.g., scheduling the work-up day, coordinating ICU/OR availability and organizing logistics) and consulted with external partners; organ allocation was performed by Eurotransplant.

Financially, all centers reported challenges covering ODEH-related costs without additional structural funding beyond standard DCD reimbursements, particularly during the early implementation of this innovative pathway. Strategies included choosing regular ambulance transport when appropriate, using existing on-call staff and equipment pragmatically, combining activities with planned capacity, and drawing on regional expertise or external support models, ultimately contributing to cost reduction.

### ODEH temporal shifts

#### Practical adjustments

Over time, teams reported practical adjustments, including a shift in one hospital from MICU to regular ambulance transport, motivated by the view that regular ambulances can provide adequate monitoring at lower cost and in a less clinical environment. Other centers continued to prefer MICU services because they were readily available within their infrastructure and simplified planning and monitoring, especially in UMCs with established MICU networks. Some teams also wore non-medical attire during home visits to create a less clinical atmosphere for relatives.

#### Ethical and regulatory challenges

Finally, ODEH raised questions about legal and regulatory frameworks, particularly regarding out-of-hospital intubation by hospital physicians and the management of an extremely low-probability “cannot intubate, cannot ventilate” scenario. Participants compared this to earlier, now settled debates on Mobile Medical Teams operating outside hospital walls and emphasized the need for clear consent documentation and case-specific and patient tailored contingency planning.

## Discussion

As of the reference date of January 2026, 13 ODEH procedures have been performed in the Netherlands since the first in 2017, and the most recent ODEH procedure occurred in the second quarter of 2025, eight of which were included in this mixed-methods evaluation [11]. Our findings show that ODEH can be conducted within the existing Dutch legal framework when core safeguards are maintained, including a clear separation between euthanasia and donation decision-making, reconfirmation of consent, and independent roles and responsibilities. In current practice, ODEH can only be pursued after an explicit patient request and when logistics can be secured; growing awareness among patients and patient organizations, together with increasing professional experience, may shift preferences towards ODEH in suitable cases. Professionals reported in multiple cases that without access to an ODEH pathway, patients would likely not have proceeded with conventional hospital-based ODE, suggesting that for some patients, ODEH constitutes an essential condition for enabling organ donation, thereby facilitating donations that would otherwise probably not have occurred. Recent Dutch transplant outcome data further support the clinical relevance of donation after euthanasia. A nationwide cohort study showed promising 10-year outcomes of kidney transplantation from donors following euthanasia, with graft outcomes comparable to other controlled donation pathways. A related commentary emphasized that these findings are scientifically encouraging while also requiring continued ethical reflection and careful governance [15-17]. Available outcome studies on liver and lung transplantation after donation following euthanasia also support feasibility, while emphasizing the importance of careful donor selection and optimal logistics; organ-specific outcome data after ODEH itself remain limited [18, 19].

ODEH’s feasibility hinges on the ability to coordinate a well-aligned, time-sensitive care pathway across home and hospital settings. In all cases, deep sedation was initiated before transfer to respect the patient’s preference for a home farewell and to avoid conscious hospital admission. However, the pathway demands careful planning and risk management, particularly around out-of-hospital airway management, transport, and alignment of ICU/OR capacity. Centre-level variation in organization (local vs. externally supported models), transport modality (regular ambulance vs. MICU), and perceived “risk” illustrates that implementation depends on local infrastructure, professional networks, and experience rather than on a single, fixed institutional model.

Despite this research, our understanding of patients’ motivations for requesting ODEH remains limited. In interviews, professionals hypothesized that autonomy and control, fear of hospital settings, previous care experiences, cultural attitudes towards death and dying and the perceived burden on relatives may all contribute, but these factors remain insufficiently understood [18]. Future research, among patients’ relatives and euthanasia providers, may clarify motivations and experiential outcomes and help ensure equitable access.

Logistical choices intersect with costs and patient experience. The reported shift from MICU to regular ambulance transport in one center reflects an attempt to “right-size” resources to anticipated risk while reducing costs and limiting the clinical footprint in the home environment. Centers that continue to use MICU services often do so because such services are readily accessible within their regional infrastructure and simplify planning and monitoring but may increase costs and can feel more “hospital-like.” The use of regular ambulance transport should therefore be interpreted primarily in terms of necessity and proportionality but also considering local accessibility and the organizing physician’s network [20]. Notably, in the absence of formal recommendations and guidelines, a dedicated NTS funding arrangement has been in place since September 2025. This funding arrangement, organized by the Dutch Ministry of Health, Welfare and Sports, covers the home-to-hospital phase, after which standard DCD donation reimbursement applies from hospital admission onwards. This structural funding may lower barriers to implementation and reduce dependence on ad-hoc solutions.

Rather than calling for rigid protocolization, our findings support developing a practical ODEH manual that specifies core conditions and decision points (e.g., role separation, consent documentation, minimum monitoring and transport requirements and coding guidance), while preserving discretionary space for tailoring to the patient’s home context, family system and local resources. This flexibility is important because ODEH is explicitly designed for the patient’s final conscious moments, and feasibility becomes clear only when an individual plan is mapped onto the home situation and regional infrastructure. The observed variability, therefore, reflects the pioneering character of redesigning an ODE pathway that starts at home while safeguarding procedural integrity [21, 22].

Ethical critiques have highlighted the potential moral burden for clinicians when euthanasia and organ donation are closely coupled [23]. ODEH may intensify this burden for some staff because of the out-of-hospital phase and prolonged coordination. Transparent role separation, team debriefing and institutional support are therefore critical. International experience remains limited but is expanding; with organ donation after medical assistance in dying or euthanasia now reported in several jurisdictions, including Belgium, Canada, Spain, Australia, New Zealand and the Netherlands [2, 22–27]. Spain has issued a national protocol for donation after assistance in dying, and Canadian experience shows that home-based MAiD followed by hospital-based lung recovery is feasible. The Dutch ODEH pathway differs by administration of the final euthanasia medication and subsequent death determination in hospital setting after prior home-based sedation, airway management and transportation.

Finally, concerns about hospital mortality indicators (e.g., HSMR/ICU metrics) appear mainly registrational. Consistent documentation and registry coding (e.g., “admission for organ donation only”) can mitigate unintended effects on institutional performance indicators, as in other donation pathways.

### Strengths and limitations

This study provides preliminary, nationwide insight into ODEH beyond single-case reports by combining anonymized case data with in-depth accounts from directly involved professionals. The mixed-methods approach allowed us to describe both what was done (case characteristics and logistical choices) and how professionals experienced the practical, ethical and legal challenges of initiating the pathway at home.

Limitations should be acknowledged. First, the sample was small (all ODEH procedures up to February 2024; n = 8) and six of eight cases were concentrated in a single hospital, limiting representativeness. National experience has expanded since then: as of January 2026, 13 ODEH cases had been completed, with broader regional distribution and implementation in both UMCs and STZ hospitals, and the most recent case occurred in the second quarter of 2025. Second, we did not interview bereaved relatives or euthanasia-providing physicians, limiting insight into patient-family experience and the emotional impact on all involved professional groups. Third, formal data saturation could not be achieved due to the small eligible pool.

Finally, observed center-level variability likely reflects the absence of a dedicated, practice-oriented ODEH manual. Our results suggest that a shared set of core conditions and decision points is feasible and desirable, while preserving flexibility for patient-centered tailoring.

## Conclusion

This mixed-methods study is the first systematic exploration of Dutch experience with ODEH, a form of end-of-life care that combines euthanasia with organ donation while enabling patients to say farewell at home. Since its introduction in 2017, 13 patients have chosen ODEH (reference date January 2026); eight early cases informed this analysis. In several cases, professionals reported that patients would not have proceeded with conventional, hospital-based ODE if a structured approach to ODEH had not been available, suggesting ODEH may enable donation that would otherwise not occur.

Our findings indicate that ODEH is feasible within the current Dutch legal framework and is highly valued by patients, relatives and professionals and preserves a home-based farewell while maintaining strict separation between euthanasia and donation trajectories. Feasibility, however, does not imply uniform ease: ODEH can be logistically and emotionally demanding and may be experienced as morally burdensome by some clinicians.

We recommend developing a practice-oriented ODEH manual that defines core safeguards and decision points without rigid protocolization, thereby supporting consistent quality while retaining room for flexible, case-specific tailoring. ODE and ODEH should be viewed as complementary options guided by the patient’s preferences and context.

## Data Availability

The data analyzed in this study are not publicly available to protect participants’ privacy and because of the potentially identifiable nature of the small number of cases. Requests regarding the datasets should be directed to the corresponding author.

## References

[1] Bollen J Vissers K van Mook W . Organ donation after euthanasia for patients suffering from Huntington's disease. J Palliat Med (2022) 25(4):527–8. 10.1089/jpm.2021.0647 35363063

[2] Mulder J Sonneveld H Van Raemdonck D Downar J Wiebe K Domínguez-Gil B Practice and challenges for organ donation after medical assistance in dying: a scoping review including the results of the first international roundtable in 2021. Am J Transpl (2022) 22(12):2759–80. 10.1111/ajt.17198 36100362 PMC10092544

[3] Maes G Oude Voshaar R Bollen J Marijnissen R . Burden of organ donation after euthanasia in patients with psychiatric disorder. BMJ Case Rep (2022) 15(7):e246754. 10.1136/bcr-2021-246754 35787499 PMC9255367

[4] van Dijk N Vliegen F Ham A de Jongh W Berkhout FJ Blankevoort J Organ donation after euthanasia in a patient living with dementia: a landmark case report. Front Dement (2023) 2:1287236. 10.3389/frdem.2023.1287236 39081971 PMC11285699

[5] Mulder J Sonneveld JPC . Organ donation following euthanasia starting at home. Transpl Int (2017) 30(10):1075–6. 10.1111/tri.13003 28667778

[6] Tajaâte N van Dijk N Pragt E Shaw D Kempener-Deguelle A de Jongh W Organ donation after euthanasia starting at home in a patient with multiple system atrophy. BMC Med Ethics (2021) 22(1):120. 10.1186/s12910-021-00686-4 34488729 PMC8422701

[7] Mulder J Sonneveld H . Organ donation after euthanasia starting with anesthesia at home is legal in the Netherlands, Belgium, Canada and Spain. BMC Med Ethics (2023) 24(1):34. 10.1186/s12910-023-00906-z 37248520 PMC10227959

[8] KNMG; KNMP. Execution of Euthanasia and Assisted Suicide. Utrecht: Royal Dutch Medical Association (2024). Available online at: https://www.knmg.nl/actueel/nieuws/nieuwsbericht/knmgknmp-richtlijn-uitvoering-euthanasie-en-hulp-bij-zelfdoding-herzien (Accessed April 20, 2025).

[9] Nederlandse Transplantatie Stichting. Richtlijn Orgaandonatie Na Euthanasie: Instructies Voor De Praktijk. Version 4.1. Leiden: Nederlandse Transplantatie Stichting (2024). Available online at: https://cdn.sanity.io/files/ca4fv15o/production/fbfff40f64ef684eedb61aa08b1220c68e1b39f3.pdf (Accessed November 10, 2025).

[10] Dutch Transplant Foundation. Richtlijnen Donatie Na Euthanasie. Leiden: Dutch Transplant Foundation. Available online at: https://transplantatiestichting.nl/protocollen/euthanasieDutch (Accessed January 4, 2026).

[11] Tajaâte N van Dijk N Pragt E Shaw D Kempener-Deguelle A Jongh Wd. Reply to reaction on ‘organ donation after euthanasia starting at home in a patient with multiple system atrophy – case report. BMC Med Ethics (2023) 24(1):33. 10.1186/s12910-023-00914-z 37248488 PMC10228023

[12] World Medical Association. Declaration of Helsinki: ethical principles for medical research involving human subjects. JAMA (2013) 310(20):2191–4. 10.1001/jama.2013.281053 24141714

[13] Netherlands. Medical research involving human subjects act (wet medisch-wetenschappelijk onderzoek met mensen—WMO). Bull Acts Decrees (Staatsblad) (1998) 161.

[14] Cleland J Durning SJ . Researching medical education. AMEE guide no. 87. Med Teach (2019) 41(2):161–73. 10.1002/9781118838983 29557693

[15] Tong A Sainsbury P Craig J . Consolidated criteria for reporting qualitative research (COREQ): a 32-item checklist for interviews and focus groups. Int J Qual Health Care (2007) 19(6):349–57. 10.1093/intqhc/mzm042 17872937

[16] Susanna C van Dijk N de Jongh W Verberght H van Mook W Bollen J Promising results of kidney transplantation from donors following euthanasia during 10-Year follow-up: a nationwide cohort study. Transpl Int (2024) 37:13142. 10.3389/ti.2024.13142 39494307 PMC11528710

[17] Jansen NE Gardiner D Results of kidney transplantation from donors following euthanasia in the Netherlands: benchmarking science and ethical challenge. Transpl Int (2024) 37:13806–24. 10.3389/ti.2024.13806 39494308 PMC11528704

[18] van Reeven M Gilbo N Monbaliu D van Leeuwen OB Porte RJ Ysebaert D Evaluation of liver graft donation after euthanasia. JAMA Surg (2020) 155(10):917–24. 10.1001/jamasurg.2020.2479 32777007 PMC7407314

[19] Ceulemans LJ Vanluyten C Monbaliu D Schotsmans P Fieuws S Vandervelde CM Lung transplant outcome following donation after euthanasia. J Heart Lung Transpl (2022) 41(6):745–54. 10.1016/j.healun.2022.01.1375 35227627

[20] Tchana-Sato V Hans G Brouckaert J Detry O Van Cleemput J Rex S Successful heart transplantation from donation after euthanasia with distant procurement using normothermic regional perfusion and cold storage. Am J Transpl (2022) 22(12):3146–9. 10.1111/ajt.17204 36131641

[21] van Dijk N Shaw D Shemie S Wiebe K van Mook W Bollen J . Directed organ donation after euthanasia. Transpl Int (2023) 36:11259. 10.3389/ti.2023.11259 37324219 PMC10262997

[22] Bollen JAM Ten Hoopen R van der Hoeven MAHBM Shaw D Brierley J Ysebaert D Organ donation after euthanasia in children: belgian and Dutch perspectives. Arch Dis Child (2019) 104(9):827–30. 10.1136/archdischild-2018-315560 30154185

[23] Middleton CA . Organ donation after MAiD: it’s not that simple. CMAJ (2019) 191(38):E1062. 10.1503/cmaj.72983 31548196 PMC6763331

[24] Ysebaert D Van Beeumen G De Greef K Squifflet JP Detry O De Roover A Organ procurement after euthanasia: belgian experience. Transpl Proc (2009) 41(2):585–6. 10.1016/j.transproceed.2008.12.025 19328932

[25] Wiebe K Wilson L Lotherington K Mills C Shemie SD Downar J Deceased organ and tissue donation after medical assistance in dying: 2023 updated guidance for policy. CMAJ (2023) 195(25):E870–E878. 10.1503/cmaj.230108 37364914 PMC10292956

[26] Organización Nacional de Trasplantes. Protocolo nacional de donación de órganos tras la aplicación de la prestación de ayuda para morir (2022). Available online at: https://www.ont.es/wp-content/uploads/2024/03/PROTOCOLO-NAC-DONACION-TRAS-APLICACION-DE-LA-PRESTACION-DE-AYUDA-PARA-MORIR.pdf (Accessed April 26, 2026).

[27] Organ Donation New Zealand. An ethics framework to support deceased donation after assisted dying in aotearoa New Zealand (2025). Available online at: https://donor.co.nz/assets/Uploads/Ethics-Framework-to-support-donation-after-assisted-dying-version-10.2.pdf (Accessed April 26, 2026).

